# Adjuvant therapy with oral sodium clodronate in locally advanced and metastatic prostate cancer: long-term overall survival results from the MRC PR04 and PR05 randomised controlled trials

**DOI:** 10.1016/S1470-2045(09)70201-3

**Published:** 2009-09-09

**Authors:** David P Dearnaley, Malcolm D Mason, Mahesh KB Parmar, Karen Sanders, Matthew R Sydes

**Affiliations:** a Royal Marsden Foundation Trust and Institute of Cancer Research, Sutton, Surrey, UK; b School of Medicine, Cardiff University, Velindre Hospital, Cardiff, UK; c MRC Clinical Trials Unit, London, UK

## Abstract

**Background:**

Bisphosphonates might modulate the development of symptomatic bone metastases in men with prostate cancer. The Medical Research Council (MRC) PR05 and PR04 randomised controlled trials assessed the use of sodium clodronate, an oral, first-generation bisphosphonate. We report the final analyses of long-term survival data with additional follow-up in both trials.

**Methods:**

311 men with metastatic disease were recruited to PR05 between 1994 and 1998, and 508 men with non-metastatic disease were recruited to PR04 from 1994 to 1997. All men were treated according to the recruiting site's standard practice at the time: for metastatic disease, all men were starting or responding to long-term hormone therapy; for non-metastatic disease, most men had radiotherapy, hormone therapy, or both. Men were randomly assigned to take four tablets per day of sodium clodronate (2080 mg) or matching placebo for up to 3 years (metastatic disease) or 5 years (non-metastatic). Long-term overall survival was assessed on an intention-to-treat basis in all men at sites in England and Wales using data from the National Health Service Information Centre, which held data for 278 of 311 men in the PR05 trial and 471 of 508 men in the PR04 trial. These studies are registered International Standardised Randomised Controlled Trials, numbers ISRCTN38477744 (PR05) and ISRCTN61384873 (PR04).

**Findings:**

Of the 278 men with metastatic disease, 258 (93%) were reported to have died. Evidence of a benefit for those with metastatic disease from use of sodium clodronate compared with placebo was seen in overall survival (hazard ratio [HR] 0·77, 95% CI 0·60–0·98; p=0·032). Of the 471 men with non-metastatic disease, 281 (60%) were reported to have died, with no evidence of improvement in overall survival with clodronate compared with placebo (HR 1·12, 0·89–1·42; p=0·94).

**Interpretation:**

Long-term data from these trials show that a first-generation bisphosphonate, sodium clodronate, improves overall survival in men with metastatic prostate cancer who are starting hormone therapy, but there is no evidence of an effect in men with non-metastatic prostate cancer.

**Funding:**

UK MRC; and an education grant and free drug from Roche Products Ltd.

## Introduction

Bisphosphonates might modulate the development of symptomatic bone metastases in men with prostate cancer. The Medical Research Council (MRC) PR05^1^ and PR04^2^ randomised controlled trials assessed the role of adjuvant sodium clodronate in men with metastatic (M1) and non-metastatic (M0) prostate cancer, respectively. Both trials have previously reported results on their primary outcome measures.^1^, ^2^

Overall survival was a secondary outcome measure in both trials, but the overall survival data were immature when the primary analyses were published. The metastatic trial, PR05, previously showed some evidence of improvement in overall survival with clodronate (hazard ratio [HR] 0·80, 95% CI 0·62–1·03); whereas the non-metastatic trial, PR04, did not show evidence of a benefit in overall survival (HR 1·02, 95% CI 0·80–1·30).

Now, 5 years after the primary outcome measure of the metastatic trial was published, we report the long-term survival data. The aim of this paper is to report final analyses of long-term survival data with additional follow-up in both trials.

## Methods

### Patients

The methods for these two trials have been fully described previously.^1^, ^2^ In summary, 311 men with bony metastases from prostate cancer (M1) and 508 men with prostate cancer without metastases (M0) gave written informed consent to join these two multicentre, double-blind, placebo-controlled randomised controlled trials, which opened to recruitment in June, 1994, and successfully completed accrual in July, 1998, and December, 1997, respectively. All sites obtained ethics approval. The trial was run under a Clinical Trial Marketing Product licence from the regulatory authority.

To view the **full protocols of these trials** see http://www.ctu.mrc.ac.uk/research_areas/study_details.aspx?s=60 and http://www.ctu.mrc.ac.uk/research_areas/study_details.aspx?s=61

### Randomisation and masking

Randomisation was done centrally using minimisation across a number of stratification factors to ensure balanced groups. In the PR05 trial in men with metastatic disease, the stratification factors were treatment centre, time since starting long-term hormone therapy (≤6 weeks *vs* >6 weeks), type of hormone therapy (monotherapy *vs* maximal androgen blockade), and WHO performance status. In the non-metastatic trial, PR04, the stratification factors were: treatment centre, tumour stage (T2 *vs* T3 *vs* T4), primary therapy (given *vs* not given), time from primary therapy to trial entry (none *vs* ≤12 months *vs* >12 months), and prostate-specific antigen (PSA) concentration at study entry (<50 ng/mL *vs* ≥50 ng/mL). Men were randomly assigned to supplement the usual treatment for their prostate cancer with four tablets each day of oral sodium clodronate (2080 mg), or a matching placebo.

### Procedures

Clodronate and placebo were provided free by Roche Products Ltd (Hertfordshire, UK), formerly Boeringher Mannheim (Lothian, UK). Men with metastatic disease were just starting or were already responding to standard treatment with hormone therapy (androgen suppression), which was maintained throughout the trial period. Men with non-metastatic disease were treated according to local standard practice, which was usually with radiotherapy, hormone therapy, or combined therapy. Trial medication was taken for a maximum of 3 years in men with metastases and 5 years in men without metastases. The primary outcome measure was the progression of symptomatic bone metastases or death from prostate cancer in the metastatic setting, or the development of symptomatic bone metastases or death from prostate cancer in the non-metastatic setting. There were no routine scans for asymptomatic bone metastases. After a primary outcome measure event, treatment was given according to standard practice at the site. Overall survival and toxicity were secondary outcome measures specified in the protocol.

**Figure 1:**
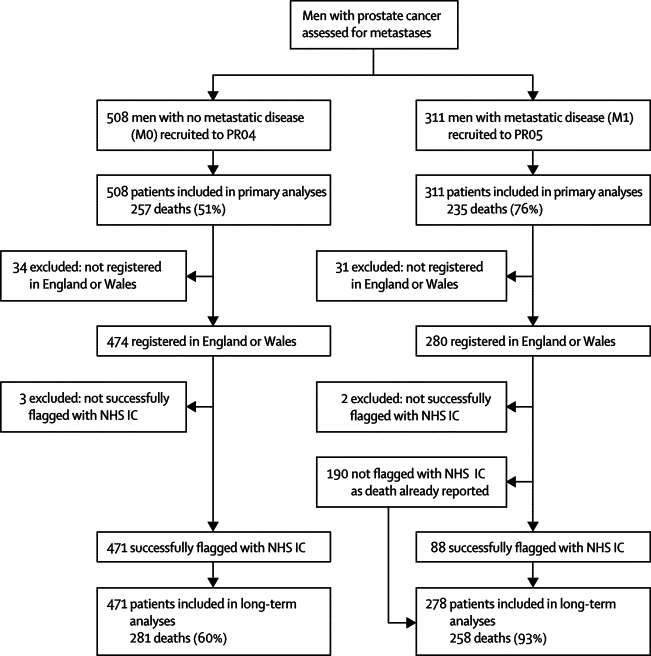
Trial profile NHS IC=NHS Information Centre (formerly Office for National Statistics). This figure shows the process of flagging and the data available. All patients on PR04 were flagged before the main, previously reported analyses, but flagging was not done in 190 patients in the PR05 trial who were already known to have died by that point. The NHS IC only provides information on patients who live in England and Wales. Therefore, 50 patients from Scotland and 15 patients from New Zealand have been excluded from these analyses. Similarly, we have excluded five patients from England and Wales who could not be matched with the NHS IC database.

For this analysis, which is only assessing long-term overall survival, we have supplemented follow-up with data from the UK National Health Service Information Centre (NHS IC), which provides data for patients from England and Wales. All patients from England and Wales who were successfully flagged are included in the analyses: 278 (89%) of 311 men with metastatic disease, and 471 (93%) of 508 men with non-metastatic disease. Patients from Scotland and New Zealand are not included in these analyses because the NHS IC does not cover these nations. Figure 1 shows the process of flagging and the data available.

### Statistical analysis

All analyses were done at the MRC Clinical Trials Unit with Stata version 10 on an intention-to-treat basis, using standard survival-analysis methods—ie, comparisons with log-rank tests and Cox proportional hazard-regression models with graphical representations using Kaplan-Meier plots. As before, we assumed that patients not reported by the NHS IC as having died when the data were locked on Sept 29, 2008, were alive 6 weeks beforehand. Exploratory interaction analyses were done using either χ^2^ tests for interaction or trend, as appropriate, to examine the consistency of the treatment effect in different subgroups; the degrees of freedom are specified in each instance. The subgroups used were the same as those used for previous analyses.^1^, ^2^ CI are given at the 95% level; p values are given to two significant figures. Since this is a planned long-term report of the survival data, no formal adjustment of p values was required. These studies are registered International Standardised Randomised Controlled Trials, numbers ISRCTN38477744 (PR05) and ISRCTN61384873 (PR04).

### Role of funding source

The sponsor and main funder of the trial had no role in the design and conduct of the trial or in the analysis of the data. Roche Products Ltd were involved in the design of the study, but not the analysis; they were invited to submit comments on an early version of this manuscript. The corresponding author had full access to the data and had final responsibility for the decision to submit the manuscript for publication.

## Results

**Figure 2:**
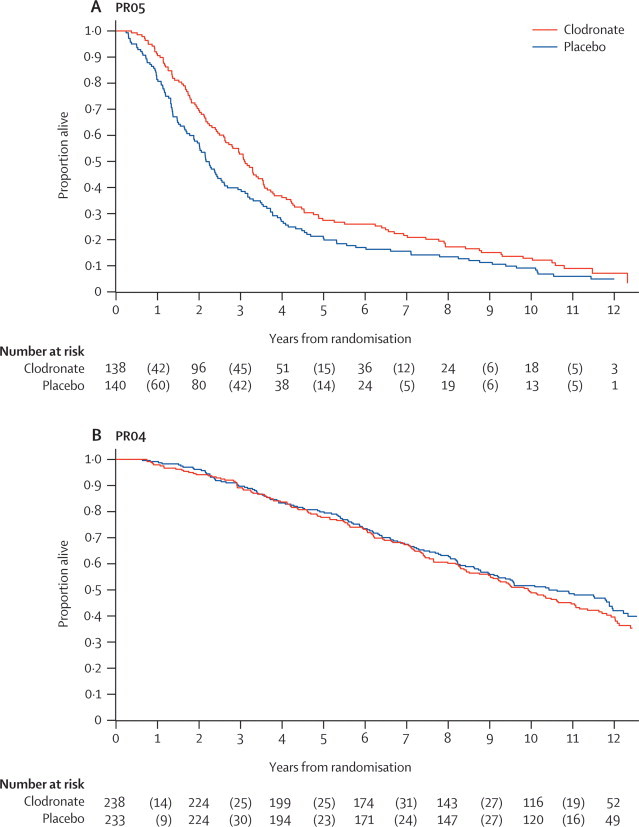
Overall survival by group in metastatic disease (PR05; A) and localised disease (PR04; B) The numbers at risk (alive) are presented at 2-yearly intervals. The numbers of events are presented in parentheses, representing the deaths during these intervals.

Over the 5-year period since the previous results, maturity in the metastatic trial, PR05, increased from 235 (76%) reported deaths in 311 men at the previously reported analyses to 258 (93%) deaths in 278 men here, with a median follow-up of 11·5 years, compared with 4·9 years previously. For overall survival, there is evidence that clodronate confers a benefit compared with placebo, with an HR of 0·77 (95% CI 0·60–0·98; p=0·032). The estimated 5-year survival was 21% with placebo and 30% with clodronate; the estimated 10-year survival was 9% with placebo and 17% with clodronate (figure 2A). In sensitivity analyses, adjusting the analyses for the stratification factors (ignoring centre) does not affect the estimate of the HR (data not shown).

In the non-metastatic trial, PR04, the number of deaths increased from 257 (51%) reported deaths in 508 men in the previously reported analyses to 281 (60%) deaths in 471 men here, with a median follow-up of 12·0 years, compared with 9·8 years previously. There is no evidence of a benefit to clodronate, with a HR of 1·12 (95% CI 0·89–1·42; p=0·94). The estimated 5-year survival was 80% with placebo and 78% with clodronate; 10-year survival rates were 51% with placebo and 48% with clodronate (figure 2B).

**Figure 3:**
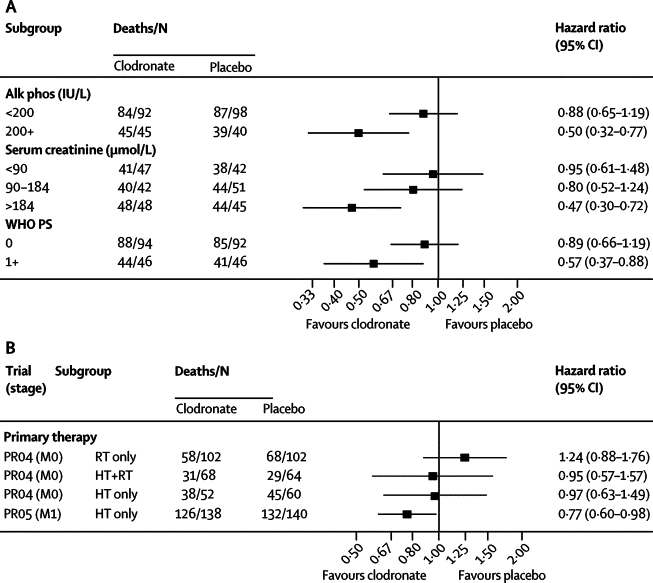
Exploratory subgroup analyses of the effect of clodronate in metastatic disease (PR05; A) and in non-metastatic disease (PR04; B), and compared with metastatic disease where all patients received hormone therapy Alk phos=alkaline phosphatase. HT=hormone therapy. RT=radiotherapy. M0=non-metastatic disease. M1=metastatic disease. WHO PS=World Health Organization performance status. (A) Alkaline phosphatase was dichotomised at a value of 200 IU/L, separating most of the values from a long tail. Serum creatinine (μmol/L) was divided into tertiles.

We did further exploratory interaction analyses for the PR05 trial in men with metastatic disease. Tests for interaction showed some evidence of heterogeneity in treatment effect on survival in subgroups defined by alkaline phosphatase (heterogeneity χ^2^=4·35, df 1; p=0·037) and serum creatinine (heterogeneity χ^2^=5·16, df 1; p=0·023; figure 3A), with larger treatment effects with higher concentrations of alkaline phosphatase and serum creatinine. Tests for interaction also showed some evidence of an increased treatment effect in men who had bone metastases for a shorter time before randomisation (heterogeneity χ^2^=3·69, df 1; p=0·055), and in men who had a shorter time from diagnosis to trial entry (heterogeneity χ^2^=3·61, df 1; p=0·058; figure 3A). There was no evidence of heterogeneity in treatment effect in subgroups defined by WHO PS (χ^2^=2·74, df 1; p=0·098), nor was there evidence of different sized treatment in subgroups defined by age at randomisation (tertiles), haemoglobin (tertiles), PSA, and time from starting hormone therapy to randomisation (data not shown).

In the PR04 trial of men with non-metastatic disease, exploratory interaction analyses focused on the choice of primary treatment, although the numbers are small in each of these groups. This analysis showed no evidence of an interaction of clodronate with primary therapy given as radiotherapy, hormone therapy, or both in terms of overall survival (figure 3B; heterogeneity χ^2^=1·13, df 2; p=0·57). The type of primary therapy administered is likely based on the underlying stage of disease. There was no evidence of heterogeneity of the treatment effect of clodronate in patients receiving clodronate with long-term hormone therapy in the metastatic trial and the non-metastatic trial (figure 3B; heterogeneity χ^2^=0·84, df 1; p=0·36).

## Discussion

Overall survival remains an important long-term outcome measure in both metastatic and non-metastatic disease. It is of clear clinical relevance, and determining the precise cause of death can be controversial. Here, we report an advantage in overall survival conferred by clodronate in men with metastases who joined the PR05 trial, but no evidence of a difference in survival in men without metastases in the PR04 trial.

These analyses considered only patients from England and Wales, which was around 90% of the patients in each trial. Patients from Scotland and New Zealand were not included in these analyses because the NHS IC does not cover these countries. The strengths of using NHS IC data are the provision of longer-term information, in which deaths are very unlikely to have been missed, and in which there is no bias in the reporting of events by centre or treatment. These benefits far outweigh the limitations of omitting the remaining patients. The choice not to go back to any centres for this analysis, and to rely on only register data, was made before these analyses were done. There is no basis for us to believe that there might be heterogeneity of treatment effect between the different countries.

Why have these two trials given apparently contradictory results—is this due to biological factors related to the development and progression of bone metastases or just the play of chance? Certainly, both trials are modestly sized, with just over 800 patients recruited in total. The power calculations were based around the previously reported primary outcome measures rather than overall survival and differences in survival might be attributable to chance, even though these analyses are based on 281 deaths in the non-metastatic setting and 258 deaths in the metastatic setting. The trial in men with metastatic disease has probably now reached its maximum feasible maturity.

However, in the metastatic setting (PR05) we have previously reported other benefits in terms of symptomatic-bone-progression-free survival with an increase in time to deterioration of performance status (HR 0·71, 95% CI 0·56–0·92) and biologically measured favourable effects on both alkaline phosphatase and PSA nadir levels in favour of clodronate therapy.^1^ Exploratory interaction analyses for the metastatic trial, reported here and previously,^1^ suggest greater relative benefit with prompt initiation of clodronate for men with poorer prognostic features such as raised alkaline phosphatase and creatinine. Patients with raised alkaline phosphatase would be expected to have increased osteoblastic activity, and we speculate that this patient population with an increased disease burden might also have a greater degree of osteoclast activation and bone lysis, which might be modified with early bisphosphonate treatment. A raised creatinine level might lead to decreased bisphosphonate excretion, and therefore relatively greater drug exposure and more biological effect.

In the trial in men with non-metastatic disease (PR04), exploratory interaction analyses focused on primary therapy, because the choice of primary therapy would have been a reflection of both disease stage and standard local practice. However, there was no evidence of an interaction between the effects of primary therapy and clodronate. For the group treated with a combination of radiotherapy and androgen deprivation, which would now be regarded as the standard of care for men with intermediate and high-risk localised disease,^3^, ^4^, ^5^ the HR was 0·95 (95% CI 0·57–1·57).

The principal action of bisphosphonates is to decrease osteoclast activity, and therefore bone resorption. Additional effects might include a secondary reduction of tumour-producing growth factors, inhibition of the adhesion of tumour cells to bone matrix, and the induction of tumour-cell apoptosis. They have an established role in the management of myeloma and metastatic breast cancer, and the treatment of hypercalcaemia related to malignancy.^6^, ^7^ Prostate cancer is characterised by osteoblastic metastases, and it remains controversial as to whether osteoclast activation is a necessary precursor for the development of these sclerotic skeletal metastases, or occurs as a consequence of their presence.^8^, ^9^, ^10^ The results of our pair of trials support the latter hypothesis, as the benefit of clodronate seemed to be restricted to the progression of established metastases. Nevertheless, the more potent amino-bisphosphonates might also have direct effects on tumour cells; inducing apoptosis and inhibiting invasion through the inhibition of the mevalonate pathway.^11^ Indeed, a recent study in localised breast cancer^12^ suggests an improvement in disease-free survival in patients treated with zoledronic acid compared with placebo (HR 0·64, 95% CI 0·46–0·91).

When the trials started in the early 1990s, chemotherapy was not routinely used in the UK to any extent to treat prostate cancer, and the options for treatment after first-line hormone therapy were very limited. The events reported in the primary outcome, based on time to newly symptomatic bone metastases, might have triggered alternative additional treatments in most cases in both trials, and these were previously summarised for the metastatic trial.^1^ There were also very few data to suggest that variations in second-line or third-line hormone treatment would make a difference to survival. We took the pragmatic decision not to collect detailed subsequent data on treatment until death, as we felt that this would be over-burdensome. There is no good reason to think there is an imbalance of these treatments across the trial groups.

In prostate cancer, trials with much more potent bisphosphonates such as zoledronic acid should help clarify the situation for men with hormone-sensitive prostate cancer in due course; a non-statistically significant trend for improved survival has already been reported using zoledronic acid in men with castrate-resistant disease.^13^ In men with hormone-sensitive disease, ongoing randomised controlled trials for men with high-risk locally advanced disease include the European Association of Urology's ZEUS trial (ISRCTN66626762) across Europe, which compares standard treatment with or without 4 mg infusions of zoledronic acid every 3 months for a total of 4 years. Additionally, the Trans-Tasman Radiation Oncology Group's RADAR trial (NCT00193856) in Australia and New Zealand, which is a four-arm trial in men having standard radiotherapy for prostate cancer, is comparing the effects on survival of duration of androgen suppression (given for either 5 or 18 months) and use of zoledronic acid (not given or given as 4 mg every 3 months for 18 months). In men with hormone-therapy-naive metastatic disease, the Cancer and Leukemia Group B CALGB-90202 trial (NCT00079001) in North America compares the effect of zoledronic acid, given as 4 mg injections every 4 weeks, with placebo. Additionally, the lead investigators from PR04 and PR05 have been involved in the design and conduct of the ongoing MRC-led STAMPEDE trial (ISRCTN78818554).^14^, ^15^ STAMPEDE is a trial for men with locally advanced or metastatic disease, and recruits similar populations to the PR04 and PR05 trials. In this six-arm trial, men starting long-term hormone therapy for the first time are randomly assigned to supplement this treatment with 4 mg zoledronic acid given intravenously every 3–4 weeks for 2 years. The other research drugs in STAMPEDE are the taxane chemotherapy docetaxel, which is given for six cycles, and the COX2 inhibitor celecoxib, which is given for 1 year. Patients in STAMPEDE are randomly assigned to receive hormone therapy alone (control group), or hormone therapy plus docetaxel; hormone therapy plus zoledronic acid; hormone therapy plus celecoxib; hormone therapy plus docetaxel plus zoledronic acid; or hormone therapy plus zoledronic acid plus celecoxib. The 2:1:1:1:1:1 randomisation means that 43% of patients are randomised to receive bisphosphonate. Between 2000 and 3000 men will join the trial; over 1000 patients have already been enrolled.

In conclusion, PR05 is the first trial, to our knowledge, to show an overall survival benefit conferred by an oral bisphosphonate when given in addition to standard hormone therapy to men with bone metastases who are starting or responding to hormone therapy for prostate cancer. However, there is no evidence that clodronate is of any benefit when given as an adjuvant to treatment in men with non-metastatic prostate cancer.
